# Phenogroup-based stratification of cardiovascular risk in obstructive sleep apnea

**DOI:** 10.1016/j.ajpc.2026.101681

**Published:** 2026-06-01

**Authors:** Anastasya Maria Kosasih, Yihui Ou, Chieh-Yang Koo, Luciano F Drager, Junjie Zhang, Rishi Sethi, Ruogu Li, Hee-Hwa Ho, Germaine Loo, Thun-How Ong, Ching-Hui Sia, Zhengfeng Chen, Hui-Wen Sim, Giap-Swee Kang, Vitaly Sorokin, Serene Wong, Nicholas Chew, Mark Y Chan, Arthur Mark Richards, Shao-Liang Chen, Huay-Cheem Tan, Ferran Barbe, Wilson W Tam, Chi-Hang Lee

**Affiliations:** a Department of Cardiology, National University Heart Centre, Singapore, Singapore; b Department of Medicine, Yong Loo Lin School of Medicine, National University of Singapore, Singapore; c Hypertension Unit-Heart Institute (InCor), University of Sao Paulo Medical School, Brazil; d Department of Cardiology, Nanjing First Hospital, Nanjing Medical University, China; e Department of Cardiology, King George's Medical University, Lucknow, India; f Department of Cardiology, Shanghai Chest Hospital, China; g Department of Cardiology, Tan Tock Seng Hospital, Singapore; h Department of Cardiology, National Heart Centre, Singapore; i Department of Respiratory and Critical Care Medicine, Singapore General Hospital, Singapore; j Department of Cardiology, Ng Teng Fong General Hospital, Singapore; k Department of Cardiac, Thoracic, and Vascular Surgery, National University Heart Centre, Singapore; l Department of Medicine, Alexandra Hospital, Singapore; m Christchurch Heart Institute, University of Otago, Dunedin, New Zealand; n Translational Research in Respiratory Medicine, University Hospital Arnau de Vilanova and Santa Maria, IRBLleida, Lleida, Spain; o Alice Lee Centre for Nursing Studies, National University of Singapore, Singapore

**Keywords:** Coronary artery disease, Phenogroup, Sleep apnea, Risk prediction

## Abstract

**Background:**

Obstructive sleep apnea (OSA) exhibits substantial phenotypic heterogeneity, but its prognostic relevance in patients with coronary artery disease (CAD) undergoing revascularization remains uncertain.

**Methods:**

We pooled data from two prospective cohorts of CAD patients undergoing percutaneous coronary intervention or coronary artery bypass grafting (*n* = 2318). Major adverse cardiac and cerebrovascular events (MACCE; cardiovascular death, non-fatal myocardial infarction, non-fatal stroke, and unplanned revascularization) were assessed over a mean follow-up of 1.9 ± 1.1 years. Latent class analysis using nine clinical variables identified phenogroups. Associations between OSA (apnea–hypopnea index ≥15) and MACCE were evaluated using Cox regression.

**Results:**

Three phenogroups were identified: (1) Younger-Sleepy-Hypoxia (42.2%), (2) Cardiorenal-Metabolic (30.9%), and (3) Old-Lean-Chinese (26.9%). OSA prevalence was similar across groups (45.5%, 50.7%, and 47.8%). The overall incidence of MACCE was 11.4% and did not differ significantly across phenogroups (*p* = 0.127). OSA was associated with increased MACCE risk in the overall cohort (adjusted HR 1.55, 95% CI 1.20–2.00; *p* < 0.001). In phenogroup analyses, this association was significant in phenogroup 1 (adjusted HR 1.57, 95% CI 1.04–2.36; *p* = 0.030), borderline in phenogroup 2 (adjusted HR 1.55, 95% CI 0.98–2.43; *p* = 0.059), and not significant in phenogroup 3 (adjusted HR 1.32, 95% CI 0.81–2.16; *p* = 0.263).

**Conclusions:**

In revascularized CAD patients, OSA was associated with increased cardiovascular risk in the overall cohort, with heterogeneity across phenogroups. These findings support phenogroup-based risk stratification, pending external validation.

## Graphical abstract (Central Illustration)

**Figure:**
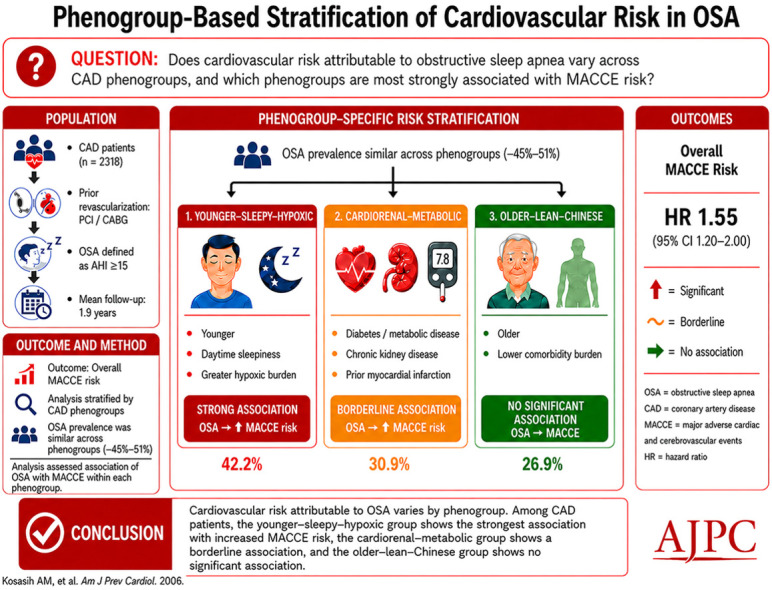

## 1 Introduction

Coronary artery disease (CAD) remains a leading cause of morbidity, mortality, and healthcare expenditure worldwide [1]. The term "CAD" encompasses a range of phenogroups based on clinical characteristics, presentations, and angiographic findings that guide different revascularization strategies [2,3]. A growing body of evidence highlights the critical role of non-traditional and modifiable risk factors in CAD outcomes, with obstructive sleep apnea (OSA) emerging as a particularly important contributor [4,5]. OSA is characterized by recurrent upper airway collapse, sympathetic hyperactivation, and nocturnal blood pressure surges, affecting nearly one billion people globally [6] and is highly prevalent in patients with CAD undergoing coronary revascularization[4,7,8]. The inconclusive results of randomized trials on continuous positive airway pressure (CPAP) therapy for improving cardiovascular outcomes have stalled progress in the field [[9], [10], [11], [12]].

OSA is now increasingly recognized as a heterogeneous disorder, with variations in symptom burden, metabolic profiles, and cardiovascular risk that may influence both disease progression and treatment response [[13], [14], [15], [16], [17]]. Recently, a post-hoc analysis showed that only a high-risk subgroup benefited from CPAP - highlighting the need to identify at-risk patients amid disease heterogeneity [18]. Moreover, emerging data from latent class and cluster analyses in sleep clinic cohorts suggest the existence of distinct OSA phenogroups, each conferring different prognostic implication [[13], [14], [15],^19^] or benefits from PAP therapy [20]. Yet, such data are largely derived from populations referred for sleep-related symptoms - limiting their relevance to cardiology practice, where OSA is often opportunistically diagnosed during risk profiling in asymptomatic patients.

To address this translational gap, we conducted a latent class analysis using two large prospective cardiovascular cohorts of patients undergoing percutaneous coronary intervention (PCI) [7] and coronary artery bypass grafting (CABG), [8] respectively. By leveraging detailed clinical and sleep-related data, we sought to identify distinct phenogroups and to evaluate how the association between OSA and major adverse cardiovascular and cerebrovascular events (MACCE) varies between these subgroups. Our findings may provide a foundation for phenogroup-driven risk stratification and more personalized approaches to managing OSA in high-risk cardiovascular populations.

## 2 Methods

This latent class analysis was a *post hoc* analysis of data from two cohort studies led by the National University Heart Centre, Singapore: the Sleep and Stent Study (NCT02215317) [21] and the SABOT study (Sleep Apnea and Bypass Operation, NCT02701504) [8]. Both studies were conducted by the same research team responsible for the present analysis, and the data were stored at the institution.

The studies were approved by the institutional review board (The Domain Specific Review Board-C: 2012/0051 and 2016/01332, respectively), and each participant provided written informed consent. Both studies were observational and did not involve any OSA-specific therapeutic interventions. The follow-up duration for each study was about two years. The primary endpoint was identical in both studies, (MACCE), defined as a composite of cardiovascular mortality, non-fatal myocardial infarction, non-fatal stroke, and unplanned revascularization. In addition, all-cause mortality was also recorded. These events were defined according to the Standardized Data Collection for Cardiovascular Trials Initiative [22]. For this latent class analysis, no additional data were collected, and study participants were not contacted. Accordingly, the institutional review board waived the requirement for a new submission.

The Sleep and Stent Study was a multicenter study (eight hospitals in five countries) evaluating the association between OSA and MACCE in patients undergoing PCI. A total of 1311 patients who had undergone successful PCI were recruited for an overnight sleep study using a US Food and Drug Administration–approved level III portable diagnostic device (Embletta Gold, Natus Medical Inc, North York, ON, Canada). Sleep study tracings were manually scored. The apnea-hypopnea index (AHI) was assessed according to the 2007 guidelines of the American Academy of Sleep Medicine [23]. Patients were classified into OSA (AHI ≥15 events/hour) and non-OSA (AHI <15 events/hour) groups. This cut-off was chosen due to the lack of cardiovascular impact of mild OSA as reported in American Thoracic Society Research Statement [24]. Apneas and hypopneas were assessed based on a ≥3% oxygen desaturation threshold, following the American Academy of Sleep Medicine's standards that were in place when the cohort was designed and the data was collected. During a median follow-up of 1.9 years (interquartile range, 0.8 years), the crude 3-year incidence of MACCE was higher in the OSA group compared with the non-OSA group (18.9% versus 14.0%; *p* = 0.001). Multivariate Cox regression analysis showed that OSA was an independent predictor of MACCE, with an adjusted hazard ratio (HR) of 1.57 (95% confidence interval (CI): 1.10–2.24, *p* = 0.013).

The SABOT study was a prospective observational cohort study conducted in Singapore. A total of 1007 patients from four public hospitals scheduled for non-emergent CABG underwent an in-hospital overnight sleep study using a US Food and Drug Administration–approved wrist-worn portable device (Watch-PAT 200, Itamar Medical, Caesarea, Israel), previously validated against in-laboratory polysomnography. The Watch-PAT 200 measures peripheral arterial tone, which is a marker of arterial pulsatile volume changes in the finger, regulated by α-adrenergic nerve activity in vascular smooth muscle. Patients were classified based on the presence or absence of OSA, defined as AHI ≥15 or <15 events/hour, respectively, and apneas hypopneas were assessed based on a ≥3% oxygen desaturation threshold. The crude 2-year incidence of MACCE was higher in the OSA group than in the non-OSA group (14.7% versus 7.8%; *p* = 0.002). Multivariate Cox regression analysis showed that OSA was an independent predictor of MACCE, with an adjusted HR of 1.57 (95% CI: 1.09–2.25, *p* = 0.015).

## 3 Latent class analysis

Latent class analysis was performed to determine clusters of clinical phenogroups. It is a clustering statistical technique that uses finite mixture modeling to classify individuals into mutually exclusive phenogroups, maximizing within-group similarities and between-group differences [25]. In this study, latent class analysis was used to identify distinct phenogroups within the 2318 study population using a data-driven, model-based clustering approach. This unsupervised statistical method enables classification based on observed covariate patterns, without reliance on predefined clinical categories, thus allowing the emergence of novel phenotypic subgroups.

We pre-specified 9 demographic and clinical variables as the latent class indicators [20]: age (categorized as ≤60 years or >60 years), sex (male or female), body mass index (BMI ≤27.5 kg/m² or >27.5 kg/m²), ethnicity (Chinese or non-Chinese), diabetes mellitus (yes or no), prior myocardial infarction (yes or no), excessive daytime sleepiness (Epworth Sleepiness Scale [ESS] >10 or ≤10), left ventricular ejection fraction (≥50% or <50%), and type of coronary revascularization (PCI or CABG). These variables were selected a priori based on their established clinical relevance to cardiovascular risk in patients with CAD and their known associations with the pathophysiology and heterogeneity of OSA. Collectively, they were chosen to capture key domains of phenotypic variation, including demographic characteristics (age, sex, ethnicity), adiposity (BMI), cardiometabolic burden (diabetes mellitus), prior cardiovascular disease (previous myocardial infarction), cardiac function (left ventricular ejection fraction), symptom burden (excessive daytime sleepiness), and clinical context of presentation (type of revascularization). All variables were consistently available across both cohorts, ensuring comparability and interpretability of the derived phenogroups.

Model estimation was conducted using Jamovi [26]. We estimated latent class models with 2–8 classes using maximum likelihood in Jamovi. Model enumeration followed a pre-specified hierarchy: statistical fit was judged primarily by the Bayesian Information Criterion (BIC) and Consistent Akaike Information Criterion (CAIC), with sample-size–adjusted BIC (SABIC), AIC, and AIC3 considered as secondary checks. These criteria balance improvement in log-likelihood against increasing model complexity; lower values indicate better fit. BIC/CAIC were prioritized because they impose stronger complexity penalties and have been shown in simulation studies to reduce over-extraction of classes in mixture models.

As a sensitivity analysis, LCA was repeated after excluding revascularization type as a class-defining indicator. Using the same indicator set and modeling specifications as the primary analysis, models with 1–8 classes were evaluated to ensure that phenogroup structure was not driven by procedural characteristics.

To assess the robustness and generalizability of the identified phenogroups, cohort-specific LCAs were additionally performed separately within the Sleep and Stent and SABOT cohorts. A cohort indicator was derived based on study enrollment identifiers, and LCAs were re-estimated within each cohort using the same indicator set and decision criteria as in the primary analysis. Model fit indices and entropy were compared across cohorts to evaluate consistency of the latent structure.

### 3.1 Statistical analysis

Baseline characteristics were compared across the three phenogroups using the Kruskal-Wallis test for continuous variables and chi-square tests for categorical variables. The incidence of MACCE was estimated using the Kaplan-Meier method, and the corresponding 2-year adverse event rates were derived from these estimates. Cox proportional hazards regression was used to examine the association between OSA and MACCE. Three models were specified: (1) an unadjusted model; (2) a minimally adjusted model including covariates not used in latent class construction (smoking, hypertension, chronic kidney disease, and cardiovascular medications [aspirin, ACE inhibitors/ARBs, beta-blockers, statins, and ezetimibe]; and (3) a fully adjusted model including these variables plus age, sex, BMI, and diabetes mellitus as a sensitivity analysis. The proportional hazards assumption was assessed using Schoenfeld residuals, with landmark analysis applied when non-proportionality was observed (early: 0-6 months and late: >6 months). Continuous oximetry and event level data were unavailable, therefore event-based hypoxic burden could not be analyzed, instead nocturnal hypoxemia indices (Time under 90% oxygen saturation (T90), oxygen desaturation index (ODI), baseline SpO2, and lowest SpO2) were evaluated descriptively and assessed for correlation as proxies of hypoxemia burden. Pairwise comparisons between phenogroups were conducted using Bonferroni-corrected post hoc tests to determine which groups differed significantly in baseline variables. A corrected *p*-value threshold of <0.0167 was applied to account for multiple testing across the three comparisons (phenogroup 1 versus 2, phenogroup 1 versus 3, and phenogroup 2 versus 3). All analyses were performed using Jamovi and Stata BE 17 [26,27].

The Sleep and Stent and SABOT data sets analyzed are not publicly available due to institutional restrictions but are available from the corresponding author on reasonable request.

## 4 Results

### 4.1 Study population

A total of 2318 patients were included in the analysis. The cohort was predominantly male (85.8%) and of Asian ethnicity (93.7%), with a mean age of 59.8 ± 9.6 years and a mean BMI of 25.5 ± 3.9 kg/m². Using the Asian BMI cut-off, 48.0% of the study cohort were classified as overweight, and 28.3% were classified as obese. The prevalence of hypertension, diabetes, and hyperlipidemia was 66.6%, 48.7%, and 68.9%, respectively. Additionally, 8.4% had a history of stroke, and 9.8% had chronic kidney disease. Use of guideline-mandated medical therapy for CAD was high and consistent with clinical practice. Sodium–glucose cotransporter 2 inhibitors, proprotein convertase subtilisin/kexin type 9 inhibitors, and glucagon-like peptide-1 agonists were not available during the time of the studies. The average AHI was 19.5 ± 17.7 events/hour, and the mean ESS score was 5.7 ± 4.2. Excessive daytime sleepiness (ESS >10) was present in 12.3% of patients. This is consistent with the prevalence (15.2%) from a post hoc analysis of three randomized trials involving patients with CAD who underwent sleep study screening[18].

The overall prevalence of OSA (AHI ≥15) in the cohort was 48.1% (1116/2318). Across the cohort, nocturnal hypoxemia indices showed similar patterns, with higher T90 and ODI and lower baseline SpO₂. Correlation analyses showed moderate to strong links between hypoxemia indices **(Supplementary Table 1)**.

### 4.2 Identification and characteristics of phenogroups

**Fig. 1:**
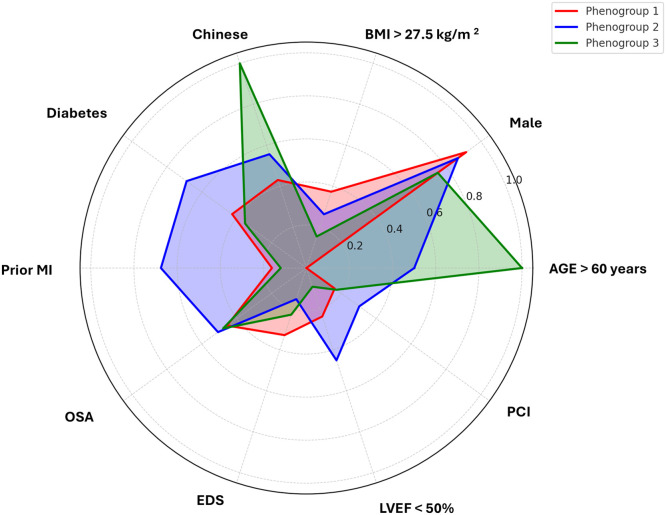
Normalized radar chart of clinical characteristics across phenogroups derived from latent class analysis.

Across models with 2–8 classes, BIC and CAIC reached their minima at the 3-class solution, identifying it as the optimal model under our primary decision rule. Although AIC and AIC3 favored a 6-class solution and SABIC favored a 5-class solution, these alternatives achieved only modest gains in log-likelihood relative to the additional parameters and yielded more complex models without clear incremental parsimony. Given the pre-specified priority for BIC and CAIC, and the aim to balance statistical fit with interpretability, we selected the 3-class model for all subsequent analyses. The entropy of the selected model was 0.473, indicating moderate classification certainty, consistent with clinical heterogeneity. These phenogroups differed notably in age, sex, BMI, ethnicity, and cardiovascular risk factor profiles (Fig. 1).

**Phenogroup 1** (42.2%, *n* = 979) comprised primarily younger and sleepy individuals (mean age: 54.0 ± 8.8 years; ESS > 10: 15.8%). Using the Asian BMI cut-off, 48.1% were classified as overweight and 37.6% as obese. This group also exhibited the longest total sleep time with SpO₂ below 90%.

**Phenogroup 2** (30.9%, *n* = 716) was characterized by a high prevalence of cardiorenal-metabolic comorbidities. The prevalence of diabetes mellitus was 68.7%, hyperlipidemia was 82.7%, and chronic kidney disease was 19.7%. This phenogroup also had the highest proportion of patients with a history of myocardial infarction (67.5%) and prior PCI (30.3%).

**Phenogroup 3** (26.9%, *n* = 623) comprised primarily older, lean Chinese patients. This group had a low prevalence of cardiovascular risk factors, relatively preserved sleep parameters, and the lowest comorbidity burden.

Details of the clinical parameters among the 3 phenogroups are shown in Table 1.

**Table 1:** Baseline demographic and clinical characteristics.

**Demographic characteristics**	Class 1 (*n* = 979)	Class 2 (*n* = 716)	Class 3 (*n* = 623)	*P* value
	**Younger-Sleepy-Hypoxia**	**Cardiorenal-Metabolic Comorbidities**	**Older, Lean Chinese**	
Age (years), mean (SD)	54.0 (8.8)	61.4 (8.1)	67.0 (6.6)	<0.001
Male sex, % (n)	91.6 (897)	86.9 (622)	75.3 (469)	<0.001
Weight (kg), mean (SD)	74.2 (12.7)	68.4 (12.2)	65.1 (9.5)	0.001
BMI (kg/m^2^), mean (SD)	26.5 (4.0)	25.4 (4.1)	24.0 (2.9)	<0.001
Neck circumference (cm), mean (SD)	39.2 (4.3)	38.6 (3.3)	37.7 (3.7)	<0.001
Waist circumference (cm), mean (SD)	95.4 (12.5)	93.9 (10.9)	89.4 (10.5)	<0.001
Systolic BP (mmHg), mean (SD)	131.0 (21.2)	126.0 (19.6)	131.7 (19.3)	<0.001
Diastolic BP (mmHg), mean (SD)	78.4 (12.9)	71.3 (11.1)	75.3 (11.2)	<0.001
LVEF, mean (SD)	52.6 (10.8)	46.9 (14.0)	57.4 (10.7)	<0.001
**Sleep**				
OSA, % (n)	45.5 (445)	50.7 (363)	47.8 (298)	0.102
Apnea-hypopnea index	18.7 (17.9)	21.4 (19.0)	18.5 (15.7)	0.009
Oxygen desaturation index	11.9 (14.8)	14.0 (16.1)	10.7 (12.6)	0.002
Baseline SpO2	94.2 (2.0)	93.9 (2.2)	93.8 (1.8)	0.001
Lowest SpO2	84.5 (7.3)	83.8 (4.8)	84.6 (5.6)	0.111
Total Time SpO2 < 90% (min)	24.4 (55.6)	13.3 (41.7)	17.0 (34.7)	<0.001
% Total Time SpO2 < 90	5.7 (12.2)	3.3 (9.6)	4.3 (8.8)	<0.001
ESS	6.2 (4.4)	5.4 (3.9)	5.2 (4.0)	<0.001
ESS > 10, % (n)	15.8 (155)	10.2 (73)	9.0 (56)	<0.001
**Ethnicity, % (n)**				
Chinese	43.0 (421)	56.6 (405)	100.0 (623)	<0.001
Malay	19.7 (193)	22.3 (160)	0.0 (0)	
Indian	27.4 (268)	14.4 (103)	0.0 (0)	
Others	9.9 (97)	6.7 (48)	0.0 (0)	
**Cardiovascular risk features, % (n)**				
Diabetes mellitus	42.6 (417)	68.7 (492)	35.2 (219)	<0.001
Previous stroke	4.9 (48)	11.5 (82)	10.4 (65)	< 0.001
Family history of coronary artery disease	10.2 (100)	7.1 (51)	5.6 (35)	0.002
Chronic kidney disease	4.0 (39)	19.7 (141)	7.2 (45)	<0.001
Hypertension	55.3 (541)	77.1 (552)	72.4 (451)	<0.001
Smoker	41.0 (401)	31.4 (225)	21.3 (133)	<0.001
Hyperlipidemia	63.5 (622)	82.7 (592)	61.3 (382)	<0.001
Previous myocardial infarction	16.0 (157)	67.5 (483)	11.9 (74)	<0.001
Previous PCI	16.2 (159)	30.3 (217)	17.2 (107)	<0.001
Previous CABG	3.3 (32)	1.3 (9)	2.1 (13)	0.023
**Medications, % (n)**				
Aspirin	98.1 (960)	90.9 (651)	94.9 (591)	<0.001
ACEi or ARB	71.5 (700)	34.9 (250)	61.8 (385)	<0.001
Thienopyridine	99.1 (970)	85.6 (613)	90.9 (566)	<0.001
Beta Blockers	82.0 (803)	88.7 (635)	80.4 (501)	<0.001
Statins	96.9 (949)	96.4 (690)	94.7 (590)	<0.001
Ezetimibe	4.6 (45)	1.7 (12)	6.1 (38)	<0.001
Fibrate	3.8 (37)	2.4 (17)	1.1 (7)	<0.001

Abbreviations: ACE: angiotensin-converting enzyme, ARB: angiotensin receptor blockers, BMI: body-mass index, CABG: coronary artery bypass surgery, ESS: Epworth sleep scale, IQR: interquartile range, LVEF: left ventricular ejection fraction, OSA: obstructive sleep apnea, PCI: percutaneous coronary intervention.

### 4.3 The prevalence of OSA across phenogroups

The prevalence of OSA was similar across phenogroups: 45.5% in phenogroup 1, 50.7% in phenogroup 2, and 47.8% in phenogroup 3. The prevalence of excessive daytime sleepiness was higher in phenogroup 1 (15.8%) than phenogroup 2 (10.2%) and phenogroup 3 (9.0%). Significant differences were noted across OSA-related parameters. Total time and percentage of time spent with SpO₂ < 90% were longest in phenogroup 1, reflecting greater cumulative nocturnal hypoxemia, while the ODI was highest in phenogroup 2.

### 4.4 Incidence of MACCE in the overall cohort and by phenogroup

Beginning from the date of coronary revascularization, the duration of follow-up was 1.9 ± 1.1 years. Among the entire cohort, the overall incidence of MACCE was 11.4% (*n* = 265). The incidence of the individual components of MACCE was as follows: cardiovascular mortality 2.5% (*n* = 58), non-fatal myocardial infarction 4.1% (*n* = 95), non-fatal stroke 3.1% (*n* = 72), and unplanned revascularization 5.6% (*n* = 130). The incidence of all-cause mortality was 4.3% (*n* = 100). Across the three phenogroups, the overall incidence of MACCE was similar (phenogroup 1: 10.3%, phenogroup 2: 13.4%, phenogroup 3: 10.9%; *p* = 0.127).

### 4.5 Association between OSA and MACCE

**Fig. 2:**
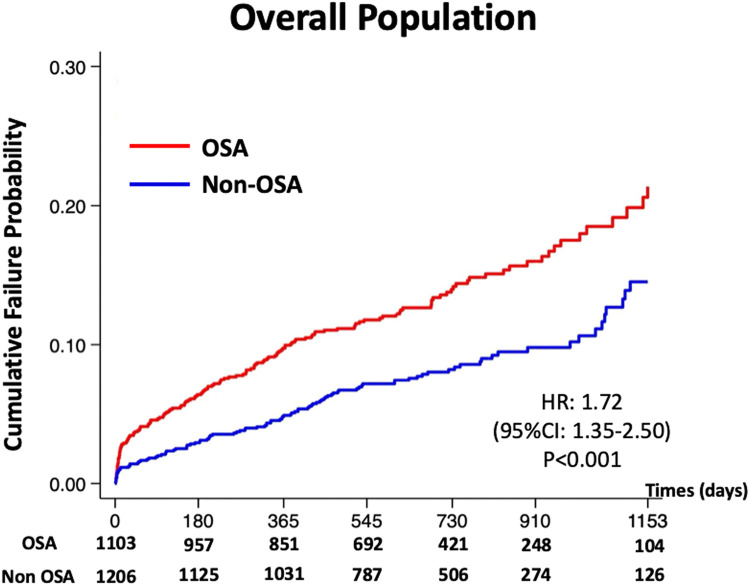
Kaplan-Meier failure curves of MACCE by OSA status in the overall cohort of 2318 patients (3 phenogroups combined). Hazard ratios in Fig. 2 are derived from unadjusted Cox models.

In the overall cohort, OSA was associated with an increased risk of MACCE (HR 1.72; 95% CI: 1.35–2.20; *p* < 0.001) (Fig. 2). Phenogroup-stratified analyses demonstrated that the crude incidence of MACCE was higher in the OSA than the non-OSA group in phenogroup 1 (2-year estimate, 13.5% versus 6.4%, HR 1.82, (95% CI 1.22–2.71), *p* = 0.003, and phenogroup 2 (15.9% versus 9.3%; HR 1.84, (95% CI 1.21–2.79), *p* = 0.004, but similar in phenogroup 3 (12.7% versus 10.1%; HR 1.40 (95% CI 0.87–2.27), *p* = 0.170.

**Fig. 3:**
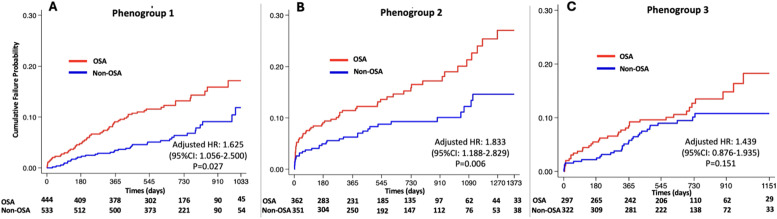
Kaplan-Meier failure curves for MACCE according to OSA status within each phenogroup. (A) Phenogroup 1, (B) Phenogroup 2, (C) Phenogroup 3.

In a minimally adjusted model including covariates not used in latent class construction (smoking, hypertension, chronic kidney disease, aspirin, ACE inhibitors/ARBs, beta-blockers, statins, and ezetimibe), OSA remained associated with MACCE in the overall cohort (adjusted HR 1.55, 95% CI 1.20–2.00; *p* < 0.001) and in phenogroup 1 (adjusted HR 1.57, 95% CI 1.04–2.36; *p* = 0.030). The association was borderline in phenogroup 2 (adjusted HR 1.55, 95% CI 0.98–2.43; *p* = 0.059) and not significant in phenogroup 3 (adjusted HR 1.32, 95% CI 0.81–2.16; *p* = 0.263) (Fig. 3). Results from the fully adjusted model were consistent and are presented as a sensitivity analysis (**Supplementary Table 2**).

In phenogroup 1, Schoenfeld residuals indicated a violation of the proportional hazards assumption for OSA. Therefore, landmark analyses were performed. During the first 6 months, OSA was significantly associated with a higher risk of MACCE compared with non-OSA (unadjusted HR 2.35; 95% CI: 1.13–4.86; *p* = 0.022). This association remained significant after adjustment for age, sex, BMI, and diabetes mellitus (adjusted HR 2.43; 95% CI: 1.13–5.23; *p* = 0.023). Beyond 6 months, OSA was associated with an increased risk of MACCE in unadjusted analysis (HR 1.62; 95% CI: 1.01–2.62; *p* = 0.047); however, this association was no longer significant after adjustment (adjusted HR 1.33; 95% CI: 0.78–2.24; *p* = 0.291).

In sex-stratified analyses of the overall cohort, OSA was associated with MACCE in men (unadjusted HR 1.74; 95% CI: 1.32–2.28; *p* < 0.001), but not in women (unadjusted HR 1.76; 95% CI: 0.98–3.14; *p* = 0.058).

### 4.6 Secondary and exploratory analyses

In phenogroup-stratified analyses, hypothesis-generating findings emerged. In phenogroup 1, OSA was associated with an increased risk of cardiovascular mortality (adjusted HR 17.50; 95% CI: 1.46–209.16; *p* = 0.020, with wide confidence intervals reflecting limited event counts), non-fatal myocardial infarction (adjusted HR 2.68; 95% CI: 1.27–5.63; *p* = 0.010), and all-cause mortality (adjusted HR 4.29; 95% CI: 1.60–11.49; *p* = 0.004) **(Supplementary Fig. 1).** These associations were not observed in phenogroups 2 or 3.

The prevalence of moderate-to-severe OSA (AHI ≥15) was similar across phenogroups; however, severe OSA (AHI ≥30) was more prevalent in phenogroup 2 (*p* = 0.030). In phenogroup 2, both AHI ≥15 and AHI ≥30 were associated with an increased risk of MACCE, whereas these associations were not significant in phenogroups 1 and 3 (**Supplementary Tables 3–4**).

### 4.7 Sensitivity and cohort-specific analyses

In sensitivity analyses excluding revascularization type as a class-defining indicator, evaluation of models with 1–8 latent classes continued to support a 3-class solution, with BIC and CAIC reaching their minima at three classes. Models with additional classes yielded only marginal improvements in likelihood at the cost of increased model complexity, supporting retention of the parsimonious 3-class structure **(Supplementary Table 5)**.

When latent class models were estimated separately within the Sleep and Stent and SABOT cohorts, a 3-class solution was independently supported in both cohorts based on BIC and CAIC. Classification entropy was comparable across cohorts (Sleep and Stent: 0.504; SABOT: 0.479), indicating similar class separation. Together, these findings demonstrate that the three-phenogroup structure emerges consistently across cohorts and is not driven by cohort pooling or procedural characteristics **(Supplementary Table 6)**.

Sensitivity analyses stratified by revascularization type showed that the association between OSA and MACCE was similar between PCI and CABG participants. There was no significant interaction between OSA and revascularization type (p for interaction = 0.610). In a fully interacted model including OSA, phenogroups, and revascularization type, a higher-order interaction was observed (p for three-way interaction = 0.034), although estimates were imprecise due to sparse event counts.

## 5 Discussion

Our findings underscore the clinical importance of phenotypic classification in OSA, particularly in patients with CAD undergoing coronary revascularization. By applying latent class analysis to two large prospective cohorts treated with PCI and CABG, we identified three phenotypic subgroups: phenogroup 1, characterized by younger-sleepy-hypoxia; phenogroup 2, characterized by cardiorenal-metabolic comorbidities; and phenogroup 3, characterized by older, lean Chinese individuals. Notably, OSA was associated with MACCE in phenogroup 1 and showed a borderline association in phenogroup 2, but not in phenogroup 3. In phenogroup 1, OSA was associated with cardiovascular mortality, non-fatal myocardial infarction, and all-cause mortality-highlighting a particularly high-risk phenogroup.

The increased risk observed in the Younger–Sleepy–Hypoxia phenotype may reflect heightened biological expression of OSA-related stress. Excessive daytime sleepiness has been linked to greater sleep fragmentation, autonomic instability, and systemic inflammatory activation. Recurrent arousals and intermittent hypoxia may amplify sympathetic surges, endothelial dysfunction, oxidative stress, and plaque instability. In younger individuals with fewer competing non-OSA comorbidities, these mechanisms may exert a more direct and measurable effect on cardiovascular risk.

In the Cardiorenal–Metabolic phenotype, vulnerability may arise from multimorbidity-driven susceptibility. Diabetes, chronic kidney disease, and prior myocardial infarction may impair vascular reserve and autonomic regulation, rendering patients less tolerant of recurrent nocturnal hypoxemia. In this context, OSA may function as an additive stressor superimposed upon an already compromised cardiovascular substrate. In contrast, the absence of a statistically significant association between OSA and MACCE in the phenogroup 3 necessitates thoughtful interpretation. It may reflect lower biological susceptibility, milder systemic consequences of OSA, or competing age-related risks that attenuate the relative contribution of sleep-disordered breathing to outcomes.

To the best of our knowledge, this is the largest latent class analysis to date examining the impact of OSA on cardiovascular outcomes in patients with CAD. Prior efforts using cluster or latent class analysis—whether based on sleep clinic registries,[14,19,20] electronic health record,[28] or prospectively collected cohort study,[29,30] have revealed clinically relevant OSA subtypes. However, these studies largely involved patients referred for suspected OSA, often with high prevalence of excessive daytime sleepiness and are not directly generalizable to cardiovascular populations in whom symptoms are less prominent and the prevalence of excessive daytime sleepiness is substantially lower.

While the association between OSA and CAD is well established, randomized trials of CPAP therapy have largely failed to demonstrate cardiovascular benefit [[31], [32], [33]]. These inconclusive results have sparked debate over whether OSA independently drives atherosclerotic risk or merely coexists with traditional risk factors. Our findings suggest that such ambiguity may stem from pathophysiologic heterogeneity: only specific subgroups, namely younger-sleepy individuals and those with a high cardiorenal–metabolic burden, appear to experience adverse cardiovascular consequences from untreated OSA. This aligns with emerging evidence that OSA exerts differential effects depending on the underlying cardiometabolic milieu [20,34,35]. Our study findings indicate that neutral CPAP trial outcomes could indicate phenotypic heterogeneity rather than an absence of biological effect, as treatment effects may be limited to specific high-risk phenogroups that were not specifically targeted in prior studies. However, as an observational study, we did not assess the impact of CPAP or other OSA-directed therapies and therefore cannot infer whether targeted treatment within these high-risk phenotypes would modify cardiovascular outcomes.

Notably, our study builds on prior work by Xu et al. ¹⁶ and others who identified a “high-risk” OSA phenogroup—characterized by obesity, hypoxia, and metabolic dysregulation—that derived cardiovascular benefit from CPAP therapy. The replication of a similar risk-enriched phenogroup in our cohort of revascularized patients strengthens the external validity of this concept. Furthermore, unlike prior studies which were drawn from sleep clinic populations, our cohort reflects the real-world cardiology setting, where patients are often asymptomatic and where OSA is diagnosed opportunistically during pre-procedural screening. This distinction enhances clinical applicability and underscores the value of phenotyping in less overtly symptomatic populations.

Our findings have several implications. First, they challenge the conventional approach of evaluating OSA treatment through unstratified trials. Prior CPAP, [11,31,32] trials may have been underpowered to detect benefit due to patient heterogeneity and lack of enrichment for high-risk phenogroups [16,36]. Future trials may consider phenogroup-based stratification as a hypothesis-generating approach, pending external validation and the development of reproducible criteria for individual-level classification.

Second, our work supports expanding the criteria for risk-based OSA screening in cardiology. Current guidelines often emphasize symptom-driven approaches, yet excessive daytime sleepiness was present in only 12.3% of our cohort. This low prevalence is consistent with other cardiovascular cohorts [11,13,32] and suggests that traditional symptom-based screening tools may miss high-risk patients. In high-acuity populations such as PCI and CABG candidates, integrating clinical phenotyping into pre-procedural evaluation may help identify individuals most likely to benefit from targeted intervention. The stability of the optimal class solution supports the interpretation that the identified phenogroups reflect underlying patient phenotypes rather than procedural characteristics.

This study demonstrates that unsupervised learning approaches can identify clinically meaningful phenogroups associated with differential cardiovascular risk in patients with OSA and CAD. While AHI remains the cornerstone of OSA diagnosis, it fails to account for variations in hypoxic burden, cardiovascular reserve, and comorbidity load - all of which may influence disease expression and treatment response [4,16,18,37]. By leveraging a 9-variable latent class model, our findings help identify phenogroups with differential cardiovascular risk attributable to OSA, despite similar overall baseline risk across groups. This aligns with the broader paradigm shift toward precision sleep medicine, in which risk prediction and therapeutic decisions are individualized based on biological and clinical profiles.

Several limitations should be acknowledged. First, the use of different diagnostic devices (portable monitor in PCI patients; WatchPAT in CABG patients) may introduce variability in AHI estimation. In addition, direct measures of arterial stiffness in CABG patients using WatchPAT (e.g., pulse wave velocity) were unavailable, hindering a formal evaluation of their possible influence on device performance. However, both tools are FDA-approved and validated against polysomnography. Second, our cohort was predominantly Asian, which may limit generalizability to non-Asian populations, though it also adds important diversity to a literature dominated by Western cohorts. However, the underlying latent structure identified through unsupervised analysis could maintain its relevance in non-Asian populations, provided that common pathophysiological mechanisms exist. Third, our findings are hypothesis-generating, as the observational design precludes conclusions regarding causality. Fourth, our study population was predominantly male, which may limit the generalizability of our findings to female patients. Finally, although latent class analysis is a powerful tool for phenotyping, it is inherently data-driven and should be validated in external cohorts. Nonetheless, the concordance of our findings with phenogroup-treatment response data from other studies strengthens their relevance and calls for prospective validation.

## 6 Conclusion

In this large cohort of patients undergoing coronary revascularization, OSA was associated with adverse cardiovascular outcomes in phenogroup 1 and showed a borderline association in phenogroup 2. These findings highlight the clinical importance of recognizing heterogeneity within OSA and support a shift toward phenogroup-guided risk stratification in cardiovascular care. Our results provide a compelling rationale for future trials targeting high-risk subgroups, such as young-sleepy individuals, through precision enrolment and tailored intervention strategies. This approach may overcome limitations of previous negative trials by focusing on patients with OSA-attributable cardiovascular risk. Beyond advancing the understanding of OSA in the context of CAD, our study adds to the growing body of evidence advocating for precision medicine in sleep and cardiovascular health. These findings highlight variability in cardiovascular risk attributable to OSA across phenogroups, rather than differences in OSA itself. Further studies are needed to validate these phenogroups and to develop clinically applicable methods for individual-level classification.

**Clinical Perspective**

What is new?

• Among patients undergoing coronary revascularization, the association between OSA and cardiovascular risk differed by phenogroup, being significant in Phenogroup 1 (Younger-Sleepy-Hypoxia) and borderline in Phenogroup 2 (Cardiorenal–Metabolic Comorbidities).

What are the clinical implications?

• These findings may help refine risk stratification in patients undergoing coronary revascularization.
• Screening for OSA may help identify individuals at higher cardiovascular risk.
• Phenogroup-based approaches may inform future strategies for targeted management, pending external validation.

Alt-text: Unlabelled box dummy alt text

## Declaration of generative AI and AI-assisted technologies in the manuscript preparation process

AI tools were used for graphical illustration and language editing.

## Funding Declaration

This study did not receive any funding support

## CRediT authorship contribution statement

**Anastasya Maria Kosasih:** Writing – review & editing, Writing – original draft, Investigation, Formal analysis, Data curation, Conceptualization. **Yihui Ou:** Writing – review & editing, Writing – original draft, Investigation, Formal analysis, Data curation, Conceptualization. **Chieh-Yang Koo:** Writing – review & editing, Writing – original draft, Supervision, Project administration, Methodology, Investigation. **Luciano F Drager:** Writing – review & editing, Writing – original draft, Supervision, Project administration, Methodology, Investigation, Conceptualization. **Junjie Zhang:** Writing – review & editing, Writing – original draft, Project administration, Methodology, Investigation. **Rishi Sethi:** Writing – review & editing, Writing – original draft, Supervision, Methodology. **Ruogu Li:** Writing – review & editing, Writing – original draft, Project administration, Methodology, Investigation. **Hee-Hwa Ho:** Writing – review & editing, Writing – original draft, Project administration, Methodology, Investigation. **Germaine Loo:** Writing – review & editing, Writing – original draft, Project administration, Methodology, Investigation. **Thun-How Ong:** Writing – review & editing, Writing – original draft, Project administration, Methodology, Investigation. **Ching-Hui Sia:** Writing – review & editing, Writing – original draft, Methodology, Investigation. **Zhengfeng Chen:** Writing – review & editing, Writing – original draft, Supervision, Methodology, Investigation. **Hui-Wen Sim:** Writing – review & editing, Writing – original draft, Methodology, Investigation. **Giap-Swee Kang:** Writing – review & editing, Writing – original draft, Investigation. **Vitaly Sorokin:** Writing – review & editing, Writing – original draft, Methodology, Investigation. **Serene Wong:** Writing – review & editing, Writing – original draft, Supervision, Investigation. **Nicholas Chew:** Writing – review & editing, Writing – original draft, Methodology, Investigation. **Mark Y Chan:** Writing – review & editing, Writing – original draft, Supervision, Investigation, Funding acquisition. **Arthur Mark Richards:** Writing – review & editing, Writing – original draft, Supervision. **Shao-Liang Chen:** Writing – review & editing, Writing – original draft, Supervision. **Huay-Cheem Tan:** Writing – review & editing, Writing – original draft, Supervision. **Ferran Barbe:** Writing – review & editing, Writing – original draft, Supervision. **Wilson W Tam:** Writing – review & editing, Writing – original draft, Supervision, Investigation, Data curation. **Chi-Hang Lee:** Writing – review & editing, Supervision, Methodology, Investigation, Funding acquisition, Conceptualization.

## Declaration of competing interest

The authors declare the following financial interests/personal relationships which may be considered as potential competing interests:

Chi-Hang Lee reports administrative support was provided by National Medical Research Council. Nil reports a relationship with Nil that includes:. Nil has patent pending to Nil. None declared If there are other authors, they declare that they have no known competing financial interests or personal relationships that could have appeared to influence the work reported in this paper.

The SABOT study was supported by the National Medical Research Council of Singapore through a Transition Award and Clinician Scientist Award (NMRC/TA/012/2012; NMRC/CSA-INV/002/2015).

The Sleep Stent study was supported by the NUHS Clinician Scientist Program and a Boston Scientific Investigator-Initiated Research Grant (ISROTH10091), with additional support for Chi-Hang Lee from NMRC (TA/NMRC/012/2012) and Luciano F. Drager from FAPESP (2012/02953-2).

## Appendix Supplementary materials

Image, application 1

Image, image 2 dummy alt text
