# Supplementary material for: Phenogroup-based stratification of cardiovascular risk in obstructive sleep apnea
Source: Am J Prev Cardiol. 2026 Jun 1;28:101681. doi: 10.1016/j.ajpc.2026.101681 (PMC13326126; doi:10.1016/j.ajpc.2026.101681)
Supplement: Supplementary file 1 [file mmc1.docx]

**Supplementary Table 1.** Correlation among nocturnal hypoxemia indices

| **Variable** | **T90** | **ODI** | **Baseline SpO2** | **Lowest SpO2** |
| --- | --- | --- | --- | --- |
| T90 (min) | 1.00 | 0.47* | -0.74* | -0.49* |
| ODI (events/h) | 0.47* | 1.00 | -0.40* | -0.62* |
| Baseline SpO2 (%) | -0.74* | -0.40* | 1.00 | 0.51* |
| Lowest SpO2 (%) | -0.49* | -0.62* | 0.51* | 1.00 |

*P value <0.001

Abbreviation: T90= Time under 90% oxygen saturation

**Supplementary Table 2.** Fully adjusted model

Fully adjusted model:

All participants: HR 1.558 (1.195 2.031), p=0.001

Phenogroup 1: HR 1.468 (0.947  2.277), p=0.086

Phenogroup 2: HR 1.659 (1.038 2.651), p=0.034

Phenogroup 3: HR 1.370 (0.823  2.280), p=0.225

**Supplementary Table 3.** Distribution of OSA Severity by AHI Thresholds Across Phenogroups

|  | **MACCE** | | | **P Value** |
| --- | --- | --- | --- | --- |
|  | **Phenogroup 1** | **Phenogroup 2** | **Phenogroup 3** |  |
| AHI > 15 | 59 (13.3%) | 60 (16.6%) | 35 (11.9%) | 0.194 |
| AHI > 30 | 25 (11.4%) | 39 (20.4%) | 16 (12.4%) | 0.025* |

*P value <0.001

**Supplementary Table 4.** AHI severity and MACCE by phenogroup

|  | **Hazard Ratio** | **95% CI** | **P Value** |
| --- | --- | --- | --- |
| **AHI > 15** | | | |
| Phenogroup 1 | 1.643 | 1.068 - 2.529 | 0.024* |
| Phenogroup 2 | 1.769 | 1.149 - 2.724 | 0.010* |
| Phenogroup 3 | 1.352 | 0.821 - 2.223 | 0.403 |
| **AHI > 30** | | | |
| Phenogroup 1 | 0.915 | 0.558 - 1.498 | 0.723 |
| Phenogroup 2 | 1.977 | 1.292 – 3.022 | 0.002* |
| Phenogroup 3 | 1.291 | 0.727 – 2.291 | 0.383 |

**Supplementary Table 5.** Model fit indices for latent class models excluding revascularization type (1–8 classes)

| Number of classes | Log-likelihood | AIC | AIC3 | BIC | SABIC | CAIC | Entropy |
| --- | --- | --- | --- | --- | --- | --- | --- |
| 1 | −10,352 | 20,720 | 20,728 | 20,766 | 20,740 | 20,774 | — |
| 2 | −10,245 | 20,524 | 20,541 | 20,622 | 20,568 | 20,639 | — |
| 3 | **−10,195** | **20,442** | **20,468** | **20,592** | **20,509** | **20,618** | **0.375** |
| 4 | −10,162 | 20,394 | 20,429 | 20,595 | 20,484 | 20,630 | 0.504 |
| 5 | −10,152 | 20,393 | 20,437 | 20,645 | 20,506 | 20,689 | 0.493 |
| 6 | −10,138 | 20,381s | 20,434 | 20,686 | 20,518 | 20,739 | 0.533 |
| 7 | −10,132 | 20,388 | 20,450 | 20,745 | 20,548 | 20,807 | 0.519 |
| s | −10,126 | 20,393 | 20,464 | 20,801 | 20,576 | 20,872 | 0.538 |

**Supplementary Table 6.** Cohort-specific model fit indices for 3-class latent class solutions

| **Cohort** | **N** | **Number of classes** | **Log-likelihood** | **AIC** | **BIC** | **CAIC** | **SABIC** | **Entropy** |
| --- | --- | --- | --- | --- | --- | --- | --- | --- |
| Sleep and Stent | 1,311 | 3 | −6,729 | 13,517 | 13,667 | 13,696 | 13,575 | 0.504 |
| SABOT | 1,007 | 3 | −4,707 | 9,466 | 9,594 | 9,620 | 9,511 | 0.479 |

Latent class analyses were performed separately within each cohort using the same indicator set and modeling specifications as the primary analysis. Model selection was guided by BIC and CAIC. Entropy reflects classification quality but was not used as the primary criterion for class selection.
