# Supplementary figures and images for: Phenogroup-based stratification of cardiovascular risk in obstructive sleep apnea
Source: Am J Prev Cardiol. 2026 Jun 1;28:101681. doi: 10.1016/j.ajpc.2026.101681 (PMC13326126; doi:10.1016/j.ajpc.2026.101681)

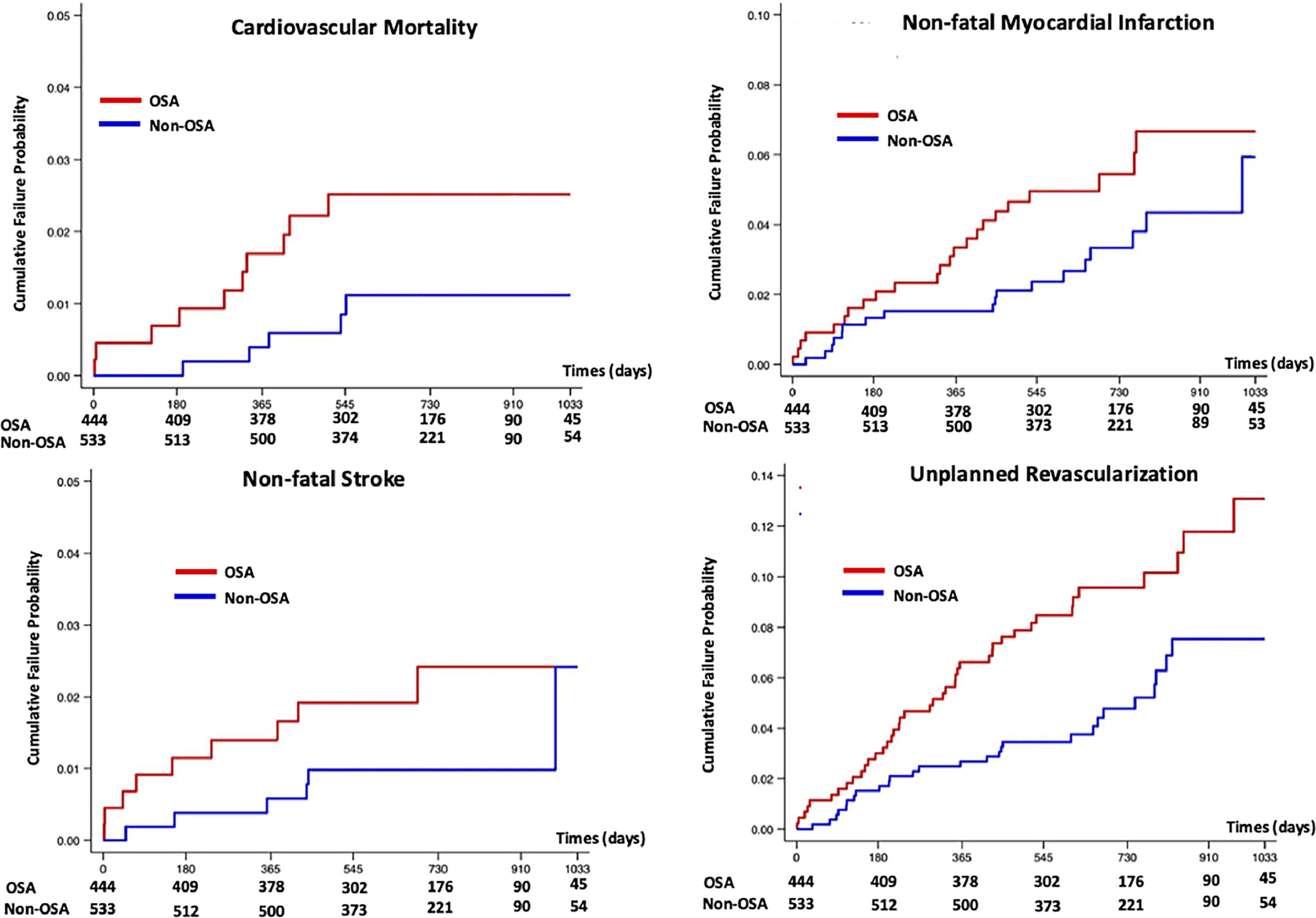

Supplement: Supplementary file 2 [file mmc2.jpg]
